# Effectiveness and applications of neurologic music therapy in motor and non-motor rehabilitation for older adults with Parkinson’s disease: a systematic review and meta-analysis

**DOI:** 10.3389/fneur.2025.1679881

**Published:** 2025-10-20

**Authors:** Aoyi Li, Yiyao Yang, Qiyu Jiang, Tiantian Wu, Tiantian Li

**Affiliations:** Wuhan Conservatory of Music, Wuhan, China

**Keywords:** neurologic music therapy (NMT), Parkinson ‘s disease, motor symptoms, non-motor symptom, geriatric rehabilitation unit, meta-analysis

## Abstract

**Purpose:**

To systematically assess the current status and effectiveness of neurologic music therapy in the rehabilitation of older adults with Parkinson’s disease.

**Materials and methods:**

A comprehensive search was conducted for randomized controlled trials. Studies were selected according to predefined inclusion and exclusion criteria. The review followed PRISMA guidelines, and methodological quality was appraised using the RoB 2.

**Results:**

Ten RCTs involving 529 older adults with PD, published mainly between 2011 and 2022, were included. Meta-analysis showed neurologic music therapy significantly improved gait velocity (SMD = 0.70, 95% CI [0.39, 1.01], *p* < 0.001) and stride length (SMD = 0.63, 95% CI [0.39, 0.88], *p* < 0.001), with moderate effect sizes, but no significant effect on cadence (SMD = 0.14, 95% CI [−0.46, 0.74], *p* = 0.65). Balance showed small-to-moderate improvement (SMD = 0.35, 95% CI [0.04, 0.66], *p* = 0.028), which became nonsignificant after sensitivity analysis (SMD = 0.29, 95% CI [−0.04, 0.62], *p* = 0.085).

**Conclusion:**

The available evidence suggests that NMT, especially RAS, shows moderate effects in improving gait speed and stride length, with relatively consistent support across studies. However, findings on cadence remain limited and are characterized by high heterogeneity. With respect to balance, pooled analyses indicated a possible mild benefit, but this effect was highly sensitive to specific studies and failed to remain statistically significant. Overall, therefore, the evidence for balance outcomes appears weak and somewhat inconsistent. With respect to quality of life and emotional well-being, the currently available quantitative evidence is both scarce and somewhat inconsistent. It can only suggest a potential benefit in a preliminary sense, and the conclusion is far from solid. More rigorously designed and higher-quality RCTs are urgently needed to confirm these findings.

## Introduction

As the global population ages at an unprecedented rate, the prevalence of neurodegenerative diseases among older adults has increased markedly. Among these conditions, Parkinson’s disease (PD) is particularly notable for its profound impact on quality of life in the elderly. PD affects approximately 1% of individuals over the age of 60, with the prevalence rising to nearly 4% in those aged 80 and above (1). As a progressive neurodegenerative disorder, PD is primarily defined by motor symptoms- including tremor, rigidity, bradykinesia, and gait disturbances- that substantially impair daily functioning and significantly reduce quality of life (2). Beyond these motor impairments, patients with PD commonly experience a range of non-motor symptoms, such as depression, anxiety, sleep disturbances, and cognitive decline, which further intensify the burden on both patients and their families. In 2021, it was estimated that 8 million people worldwide were living with PD, and this number is expected to approach 10 million by 2030 due to ongoing demographic shifts (3). As the proportion of older adults continues to grow, the prevalence of PD is anticipated to rise in parallel. This escalating trend not only heightens the economic and caregiving demands faced by patients and their families, but also presents substantial challenges for healthcare resource allocation and public health infrastructure. Considering that older PD patients differ from younger counterparts in terms of comorbidity burden, sensory decline, frailty, and polypharmacy, factors that may influence rhythmic responsiveness, the safety window of training, dose adjustments, and adherence, this review restricted the study population to individuals aged ≥60 years, in order to enhance the external validity and safety relevance of the evidence for clinical practice.

Conventional rehabilitation for Parkinson’s disease (PD) predominantly involves pharmacological treatment and physical therapy. Nonetheless, medication efficacy is frequently undermined by variable responses and adverse effects such as dyskinesia, while the long-term impact of physical therapy is often constrained by suboptimal patient adherence, limiting its sustained benefits (4). This underscores the necessity for innovative rehabilitation approaches that synergize with pharmacological regimens to comprehensively improve both motor and non-motor outcomes. Neurologic Music Therapy (NMT), a recently developed non-pharmacological intervention, has shown distinct rehabilitative benefits. Through rhythmic auditory stimulation and related modalities, NMT can activate intact neural pathways, thereby facilitating improvements in gait and motor coordination among individuals with PD (5). Furthermore, the intrinsically engaging and emotionally meaningful qualities of music interventions not only promote patient engagement but also address the persistent challenge of limited adherence encountered in conventional physical therapy.

NMT is a rigorously evidence-based intervention informed by the mechanisms of neuroplasticity and multisensory integration. This approach employs structured musical components—including rhythm, melody, and dynamic patterns—to precisely modulate central nervous system activity. Within the field of Parkinson’s disease rehabilitation for older adults, NMT is notable for its robust theoretical framework, positing that musical rhythm can modulate the basal ganglia—thalamocortical circuits and thus mitigate motor dysfunction caused by impaired neural transmission. In addition, the inherently multimodal character of music-based interventions—such as the simultaneous activation of auditory and motor networks—facilitates neuroplastic processes and supports the restoration of both motor and cognitive functions. Taken together, these features position NMT as a novel and integrative therapeutic strategy for the rehabilitation of elderly individuals with Parkinson’s disease (6).

Over the past decade, the application of NMT in the rehabilitation of Parkinson’s disease (PD) has attracted considerable attention. Emerging research indicates that music-based interventions can significantly enhance motor functions—particularly gait and balance—while also exerting positive effects on communication, swallowing ability, and emotional well-being (7). With respect to motor improvement, rhythmic auditory stimulation (RAS) has been demonstrated to effectively optimize gait parameters in individuals with PD. Systematic training has been shown to yield substantial improvements in gait velocity, stride length, and other spatiotemporal characteristics, as well as better balance and a reduced risk of falls (6, 8, 9). Thaut et al. reported that RAS gait training can significantly increase gait speed and stride length in people with PD, and improve related electromyographic (EMG) patterns (10). Pohl et al. conducted a parallel-group randomized controlled trial to evaluate the efficacy of group music interventions in PD. Their findings suggest that music intervention may enhance patients’ mood, alertness, and quality of life, although no significant differences were observed between the intervention and control groups in dual-task performance, cognitive function, balance, or freezing of gait (11). Harrison et al. compared the gait performance of PD patients during self-initiated singing (internal cueing) versus external musical cueing, finding that singing was associated with a greater reduction in gait variability. In other words, “matching one’s steps to one’s own voice” was more effective in stabilizing gait than relying solely on external musical beats (12). Similarly, Satoh et al. demonstrated that synchronized humming during walking can improve gait stability and turning fluidity in individuals with PD (13). Taken together, these findings suggest that therapeutic singing, as a form of internal rhythmic cueing, holds promise as an innovative and effective approach to gait training. Although current studies to some extent indicate that NMT holds promise across several motor and non-motor domains, the evidence is not uniformly positive. A number of randomized controlled trials or mixed-methods studies have reported no significant between-group differences, or mixed results, in outcomes such as balance, emotional/cognitive scales, and more complex gait tasks (e.g., dual-task gait, freezing of gait). For instance, Pohl et al. (11), using a group-based music intervention with a mixed design, did not observe significant improvements in some secondary outcomes. Some examples suggest that results may be influenced by multiple methodological factors, including intervention targets, control conditions, dosage and duration, the degree of rhythmic individualization, and the choice of outcome measures. Therefore, future studies should pay closer attention to stratification of patient subgroups, more rigorous control designs, and the standardization of assessment tools, in order to enhance the reliability and consistency of findings.

In the area of speech and other non-motor symptoms, growing evidence suggests that NMT provides benefits for individuals with Parkinson’s disease (PD) that extend well beyond motor function enhancement, delivering meaningful improvements across a spectrum of non-motor domains. Engaging patients in singing and vocal exercises has been shown to increase speech loudness and strengthen respiratory control, while also facilitating swallowing, alleviating emotional distress, and enhancing overall quality of life (14). For instance, Stegemöller and colleagues reported that after an eight-week singing intervention, participants with PD exhibited significant gains in maximum inspiratory and expiratory pressures, as well as in maximum phonation time, alongside subjective improvements in voice and life quality scores (15). In another pivotal study, Pacchetti et al. demonstrated that a three-month group percussion training program led to not only reduced bradykinesia but also enhanced communication skills, collaboration, and cognitive function (16). However, current findings are marked by substantial heterogeneity, with notable variability in intervention designs and methodologies, as well as the absence of standardized outcome measures.

To date, there is a notable scarcity of comprehensive systematic reviews and meta-analysis focused on older adults (≥60 years) with Parkinson’s disease (PD), especially those that incorporate the most recent research developments (14, 17). Importantly, most English-language reviews rarely include studies from China, largely due to linguistic barriers and related challenges. Considering that China represents 18.1% of the global population according to the 2021 census, it is imperative that evidence from Chinese research be integrated into worldwide systematic assessments. Given that previous English-language reviews have rarely included Chinese studies in a systematic manner, we consider the integration of Chinese evidence to be highly valuable. However, the present systematic review was conducted on peer-reviewed studies published in English-language journals, in order to avoid difficulties for international readers in accessing and interpreting Chinese-language sources. Nevertheless, several studies based on Chinese patients with Parkinson’s disease are still represented. It should be emphasized that the omission of a systematic search of Chinese databases constitutes an important evidence gap and a key limitation of this review. Future work should address this issue, for example, by conducting a comprehensive review covering CNKI, Wanfang, and VIP databases. Given the rapid progression of population aging and the high prevalence of PD among older adults, a targeted systematic review of interventions for this group is of significant academic and practical value. Furthermore, many of the relevant studies utilize randomized controlled trial (RCT) methodologies, recognized as the gold standard in clinical research. Synthesizing the results from these RCTs is essential for providing high-quality evidence to inform clinical practice and shape future research agendas.

This review provides a comprehensive synthesis of English-language studies published from 1996 to 2025 that examine the use of NMT in the rehabilitation of older adults with Parkinson’s disease. Adhering to the PRISMA 2020 guidelines, this review aims to: (1) systematically identify randomized controlled trials (RCTs) of NMT involving PD patients aged 60 years or older; (2) critically evaluate the impact of NMT on both motor symptoms (including gait, balance, and motor performance) and non-motor symptoms (such as quality of life, cognitive function, and emotional well-being); and (3) provide evidence-based recommendations to inform clinical practice and guide future research in this domain.

## Materials and methods

This systematic review and meta-analysis was conducted and reported in full compliance with the PRISMA 2020 guidelines for systematic reviews and meta-analyses (18). To uphold methodological rigor and transparency in the inclusion process, we adopted the following procedures.

### Study design and framework

This review adopts the PICO framework to provide a systematic evaluation of the functional outcomes, health benefits, and therapeutic impact of Neurologic Music Therapy (NMT) in older adults with Parkinson’s disease. Eligible participants were individuals with a confirmed diagnosis of PD aged 60 years or above. The interventions assessed included NMT and its specific modalities, such as rhythmic auditory stimulation and vocal training. Comparator groups consisted of standard rehabilitation, placebo interventions, or absence of intervention. The primary outcomes focused on motor function—including gait, balance, and scores on the Unified Parkinson’s Disease Rating Scale Part III (UPDRS-III)—while secondary outcomes encompassed cognitive performance, emotional well-being, quality of life, and safety. The International Classification of Functioning, Disability and Health (ICF) developed by the World Health Organization served as the principal analytical framework for evaluating and coding health outcomes following NMT interventions. A detailed description of the PICO framework employed in this review is provided in Table 1. Two independent coders, following a pre-specified ICF coding manual, mapped all scales and objective measures to the second-level ICF codes. Inter-rater agreement was tested using Cohen’s kappa coefficient. In cases of disagreement, the two coders first discussed the issue; if consensus could not be reached, a third senior reviewer served as arbiter, with the rationale for arbitration fully documented. Agreement analysis was conducted using Cohen’s kappa coefficient with 95% confidence intervals. The estimated *κ* values ranged from 0.73 to 0.85, indicating a high level of inter-rater agreement, which, according to the thresholds proposed by Landis & Koch, can be classified as “substantial.”

**Table 1:** The PICO framework of this study.

Population	Intervention	Comparison	Outcome
**Diseases and functional Impairments**	**Types of intervention**	Pre- and post-intervention comparison	**Motor function**
*Decline in walking ability*	*Rhythmic auditory stimulation*	Comparison of different modes of intervention	*d450 Improvement in walking ability*
*Abnormality in muscle Tone function*	*Patterned sensory enhancement*	Control group receiving non-music therapy	*b770 Improvement in gait pattern functions and balance*
*Impaired voluntary motor control*	*Therapeutic instrumental music performance (TIMP)*		**Cognitive functions**
*Decline in attention and memory*	*Therapeutic singing*		*b164, b144 Improvement in cognitive processing and memory*
*Depressed mood and abnormal emotional functioning*	**Intervention protocol**		**Emotional and psychological aspects**
*Decline in ability to perform activities of daily living*	*Mode of intervention*		*b152 Improvement in emotional functions, including reduction of depression and anxiety*
*Reduced participation in community life*	*Frequency of intervention*		**Quality of life and participation**
**Demographic indicators**	*Duration of intervention*		*d230 Improvement in carrying out daily routine*
*Age ≥ 60 years*			*d920 Increase in social and leisure participation*

The bolded terms are the category headings for each column.

### Data sources and search strategy

A comprehensive literature search was conducted for studies published from January 1, 1996 to April 30, 2025 using PubMed, ProQuest, and Web of Science databases. The search strategy combined subject headings and free-text keywords related to Parkinson’s disease, music therapy, and neurologic music therapy. Multiple sets of English search terms were flexibly assembled—for example, pairing “Parkinson” or “Parkinson’s disease” with “music therapy,” “neurologic music therapy,” or “rhythmic auditory stimulation”—and supplemented with age-related terms such as “elderly” and “≥60 years.” Boolean operators and truncation symbols were utilized to maximize search sensitivity and specificity. To ensure the breadth of coverage, reference lists of the included studies were manually screened for additional relevant publications. The search approach was adapted to the unique indexing and functionalities of each database. Reference management software was employed for automated deduplication, thereby enhancing the methodological rigor and transparency of the literature selection process. This review complies with PRISMA 2020, and the completed PRISMA checklist is presented in Table 2.

**Table 2:** PRISMA 2020 checklist with cross-references to this review.

Section and topic	Item #	Checklist item	Location / Evidence in article
Title			
Title	1	Title indicates a systematic review/meta-analysis	Title page
Methods			
Search strategy	7	Reproducible search strategy / Date	Data Sources and Search Strategy
Selection process	8	Screening process, number of reviewers / criteria	Study Selection and Data Extraction (Three-reviewer consensus process)
Data collection process	9	Data extraction method and cross-checking	Study Selection and Data Extraction
Data items	10a/10b	Definition of variables / outcomes; handling of missing data	Eligibility / Outcomes, Characteristics of Included Studies
Study risk of bias assessment	11	Tools, reviewers, and disagreement resolution	Risk-of-Bias Assessment (RoB 2; dual-reviewer consensus)
Effect measures	12	Effect size (SMD/MD/OR, etc.)	Effects of the interventions… (forest plots and explanation of SMD)
Synthesis methods	13a–f	Synthesis model, heterogeneity, and sensitivity	Results section – forest plots for each outcome (random-effects, I^2^, sensitivity)
Reporting bias assessment	14	Assessment of reporting bias (e.g., selective reporting/publication bias)	Risk-of-Bias Assessment
Certainty assessment	15	Certainty of evidence (e.g., GRADE)	N/A
Results			
Study selection	16a/b	PRISMA flow diagram and reasons for exclusion	Figure 1 PRISMA flow diagram, Study Selection
Study characteristics	17	Table of study characteristics	Table 3 and Characteristics of Included Studies
Risk of bias in studies	18	Risk-of-bias figures and results for individual studies	Figures 2, 3 and Summary of Risk of Bias
Results of individual studies	19	Effect estimates and plots of individual studies	Figures 4, 5 and corresponding text
Synthesis results	20a-d	Pooled effects, heterogeneity, and sensitivity	Figures 4, 5 section (SMD, I^2^, sensitivity)
Reporting biases	21	Results of reporting bias assessment	Summary of Risk of Bias
Certainty of evidence	22	Certainty of evidence	Strength of Evidence for Motor Outcomes, and Strength of Evidence for Non-Motor Outcomes
Discussion			
General interpretation	23a	Interpretation of main findings	Discussion—Main Findings
Limitations of evidence	23b	Limitations at the level of primary studies	Limitations (small sample size, difficulties with blinding, heterogeneity)
Limitations of review	23c	Limitations at the level of this review	Limitations (database coverage, scarcity of non-motor evidence)
Implications	23d	Implications for practice and research	Clinical Significance + Conclusion
Other information			
Registration & protocol	24a-c	Availability of registration/protocol	not registered
Support	25	Funding sources	Funding
Competing interests	26	Conflicts of interest	Disclosure of interest
Availability of materials	27	Availability of data, code, and materials	N/A

Search example: For Web of Science, a search string was: TS = ((Parkinson* OR “Parkinson disease” OR “Parkinson’s disease”) AND (“music therap*” OR (“neurologic*” NEAR/3 “music” NEAR/3 therap*) OR “rhythmic auditory stimulation” OR “patterned sensory enhancement” OR “therapeutic instrumental music performance” OR singing OR “vocal training” OR MUSTIM OR “melodic intonation therap*”) AND (elder* OR “older adult*” OR “older people” OR “older person*” OR geriatric* OR senior*)).

This review focused on randomized controlled trials (RCTs) involving older adults (≥60 years) with Parkinson’s disease, aiming to systematically integrate evidence on Neurologic Music Therapy (NMT) and Rhythmic Auditory Stimulation (RAS). Given that the topic spans both medical and rehabilitation fields and emphasizes controlled clinical designs, we prioritized PubMed and the Web of Science Core Collection to ensure coverage of core medical and rehabilitation literature. We also included ProQuest to capture cross-disciplinary rehabilitation research and reports within the social and behavioral sciences. Considering the substantial overlap in coverage between Scopus and WoS, and the fact that Embase is primarily oriented toward pharmacological literature, while our study focused on non-pharmacological rhythm- and music-based interventions, Embase, PsycINFO, and Scopus were not included in the main search strategy.

### Eligibility criterion

Informed by the PICOS framework, this study formulated explicit inclusion and exclusion criteria to guide the selection of relevant literature, specifically targeting studies on the use of NMT for rehabilitating older adults with Parkinson’s disease.

1) **Participants**: Eligible participants were required to have a definitive diagnosis of Parkinson’s disease and be at least 60 years old. In studies with mixed-age cohorts, inclusion was permitted if data specific to individuals aged 60 or older were available, or if the mean age of the sample was no less than 60 years. Studies were included irrespective of participant living arrangements (community-dwelling or institutionalized) or disease stage. Conversely, research exclusively targeting adolescents or cases of early-onset Parkinson’s disease was excluded.
2) **Interventions:** This review included all rehabilitation interventions utilizing NMT techniques, encompassing rhythmic auditory stimulation (RAS), music-assisted movement or exercise, therapeutic instrumental performance, patterned sensory enhancement, and singing-based therapies (such as speech, vocal, and respiratory training). Additionally, other music-based cognitive or motor training modalities aligned with established NMT principles were considered. General music therapy programs were deemed eligible only when they clearly integrated specific NMT techniques or principles.
3) **Comparators:** Control groups included standard care, placebo or sham interventions, and alternative therapies such as conventional physical, occupational, or speech therapy, as well as interventions lacking a musical component. Studies employing either parallel-group or crossover randomized controlled trial (RCT) designs with a defined control condition were considered eligible for inclusion.
4) **Outcomes:** Eligible studies were required to evaluate motor outcomes—such as gait velocity, stride length, balance metrics, UPDRS-III motor scores, the Timed Up and Go (TUG) test, or the 6-Minute Walk Test—as well as non-motor outcomes, including cognitive performance, speech intelligibility, vocal intensity, mood (depression or anxiety) scales, or quality of life assessments. At least one quantitative outcome had to be reported.
5) **Study Design:** Only randomized controlled trials (RCTs) were eligible for inclusion; case series and single-case designs were excluded. Data sources were limited to studies published in peer-reviewed English-language journals. Conference abstracts without complete datasets, duplicate reports of the same study, and research in which the intervention lacked a clear musical or rhythmic component (e.g., conventional exercise training without musical cues) were not considered.

**Exclusion Criteria:** Studies that did not fulfill the above criteria were excluded. Specifically, exclusion criteria encompassed the following: participants under 60 years of age; interventions described generically as “music therapy” without explicit identification of NMT techniques (unless supplemental data verified the use of NMT methods); observational or cohort studies without a control group; non-English publications; and review articles. In cases where multiple reports were derived from the same cohort, only the most comprehensive dataset was included, with other reports serving only as sources of supplementary information.

### Study selection and data extraction

The literature selection proceeded as follows. A systematic search was first performed across major databases according to a prespecified search strategy, and all identified records were imported into EndNote for management. Titles, abstracts, and keywords were then screened in line with the inclusion criteria. At the title/abstract stage, a liberal strategy was applied: if any reviewer marked a record as “include” or “uncertain” it was moved forward to full-text review; only when all reviewers marked a record as “exclude” was it removed. Following this preliminary screening, duplicate entries were identified and removed through study characteristic comparison. Full texts were obtained for articles meeting the initial criteria, and a secondary screening excluded publications with inappropriate types or mismatched outcome measures. Reference lists of included studies were further examined to capture any potentially relevant articles missed during the initial search. Three independent reviewers conducted all screening steps, reaching consensus through cross-verification. Upon completion of study selection, two reviewers independently extracted and entered data, capturing information such as first author, country or region, year of publication, journal, sample size, participant characteristics (including age, sex, Parkinson’s disease duration and severity), intervention details (content, duration, frequency, and length), outcome measures, and assessment methods. All extracted data were cross-checked by both reviewers to ensure accuracy and consistency. Studies lacking essential data or information were excluded from the meta-analysis but were still retained in the overall systematic review. Studies that did not report means and measures of dispersion (SD/SE), or from which quantitative data could not be extracted from the text or figures, were retained within the scope of the systematic review but were not included in the meta-analysis for the corresponding outcome. Agreement among reviewers regarding screening of titles and abstracts yielded a Cohen’s kappa of 0.58 (CI: 0.40–0.77).

### Risk-of-bias assessment

The methodological quality of included randomized controlled trials (RCTs) was evaluated using the second edition of the Cochrane Risk of Bias tool (RoB 2). Two reviewers independently assessed each study across five domains: the randomization process, deviations from intended interventions (performance bias), completeness of outcome data (attrition bias), measurement of outcomes (detection bias), and selective reporting (reporting bias). Consistent with the Cochrane Handbook, each domain was rated as “low risk,” “some concerns,” or “high risk.” All risk-of-bias assessments were conducted independently by two reviewers, who provided judgments at the study level, domain level, and overall rating. In cases of disagreement, a structured consensus discussion was first undertaken; if consensus could not be reached, a third senior reviewer acted as arbiter, with detailed documentation of the rationale for arbitration. Following Cochrane guidelines, any studies deemed to be at high risk of bias were planned to be excluded from the meta-analysis but retained for supplementary discussion (19).

### Statistical analysis

Given that the number of included studies for each outcome generally did not exceed six, and that heterogeneity for gait speed and stride length was nearly 0%, we refrained from formal meta-regression to avoid low statistical power and inflated risk of multiple comparisons. For outcomes with higher heterogeneity (e.g., cadence), we predefined and reported sensitivity analyses and qualitative subgroup explorations (covering intervention targets, stimulation type, dosage and duration, disease stage, control conditions, and outcome scales). These results are intended for interpretive reference only, rather than as the basis for definitive conclusions.

## Results

### Study selection

**Figure 1:**
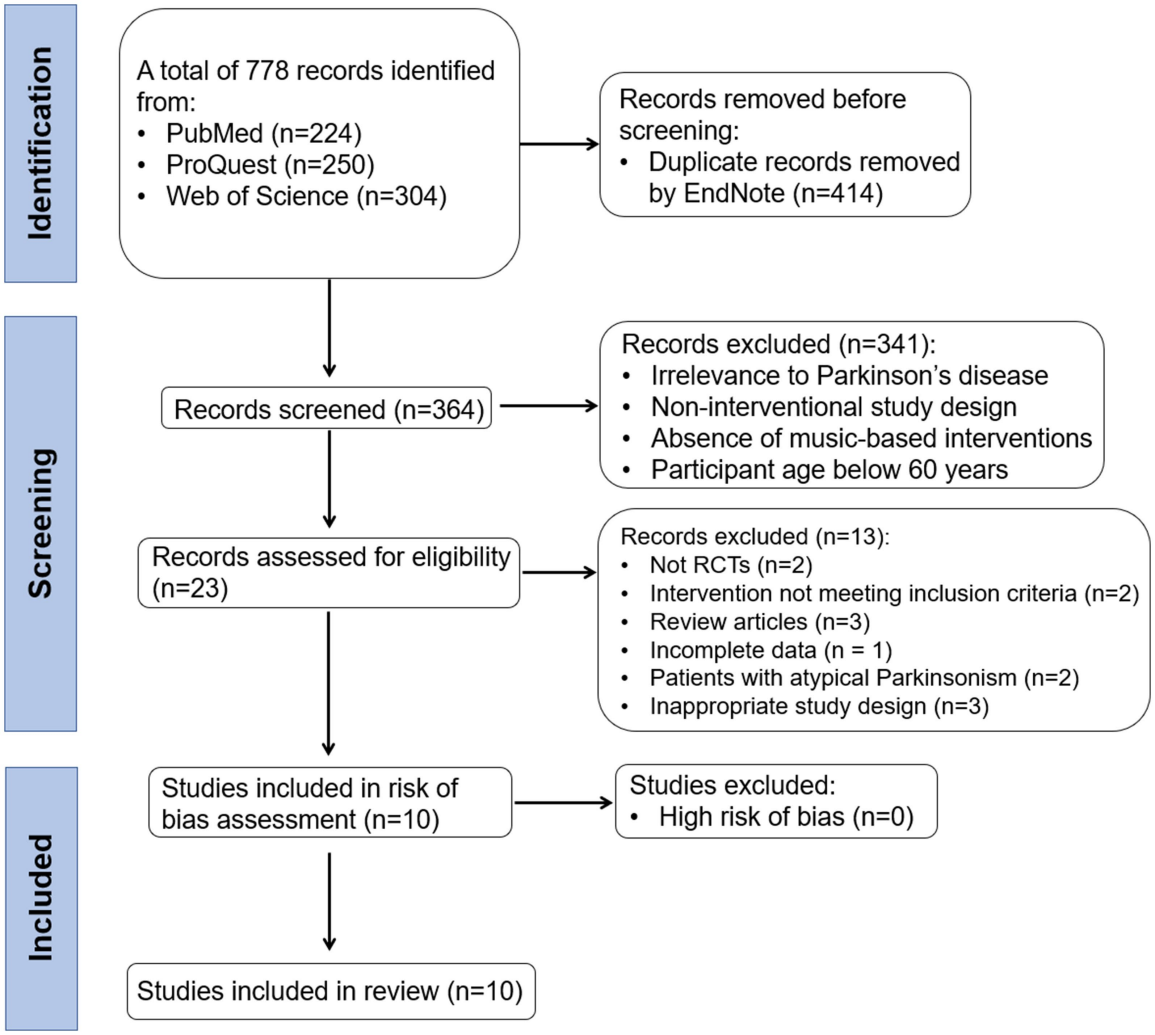
PRISMA flowchart of screening strategy for studies to be included in the review and meta-analyses. Ten RCTs were included in the systematic review and meta-analysis. Reasons for exclusion are provided at each node of the flow diagram.

A total of 778 records were identified through database searches (see Figure 1). After removing duplicates, 364 unique records remained. During title and abstract screening, 341 records were excluded for reasons such as irrelevance to Parkinson’s disease, non-interventional study design, absence of music-related content, or participant age below 60 years. Subsequently, 23 articles were selected for full-text review. Of these, 13 were excluded due to the following: lack of randomized controlled trial (RCT) design (*n* = 2), interventions not meeting inclusion criteria (*n* = 2), review articles (*n* = 3), incomplete data (*n* = 1), inclusion of atypical Parkinson’s disease patients (*n* = 2), or non-compliant study design (*n* = 3). The remaining 10 studies underwent risk of bias assessment, with none rated as high risk; therefore, all were included in the final synthesis. In sum, 10 studies fulfilled all eligibility criteria and were included in this systematic review and meta-analysis. Figure 1 illustrates the PRISMA flow diagram, which outlines the process of literature identification, screening, and final inclusion.

### Summary of risk of bias

**Figure 2:**
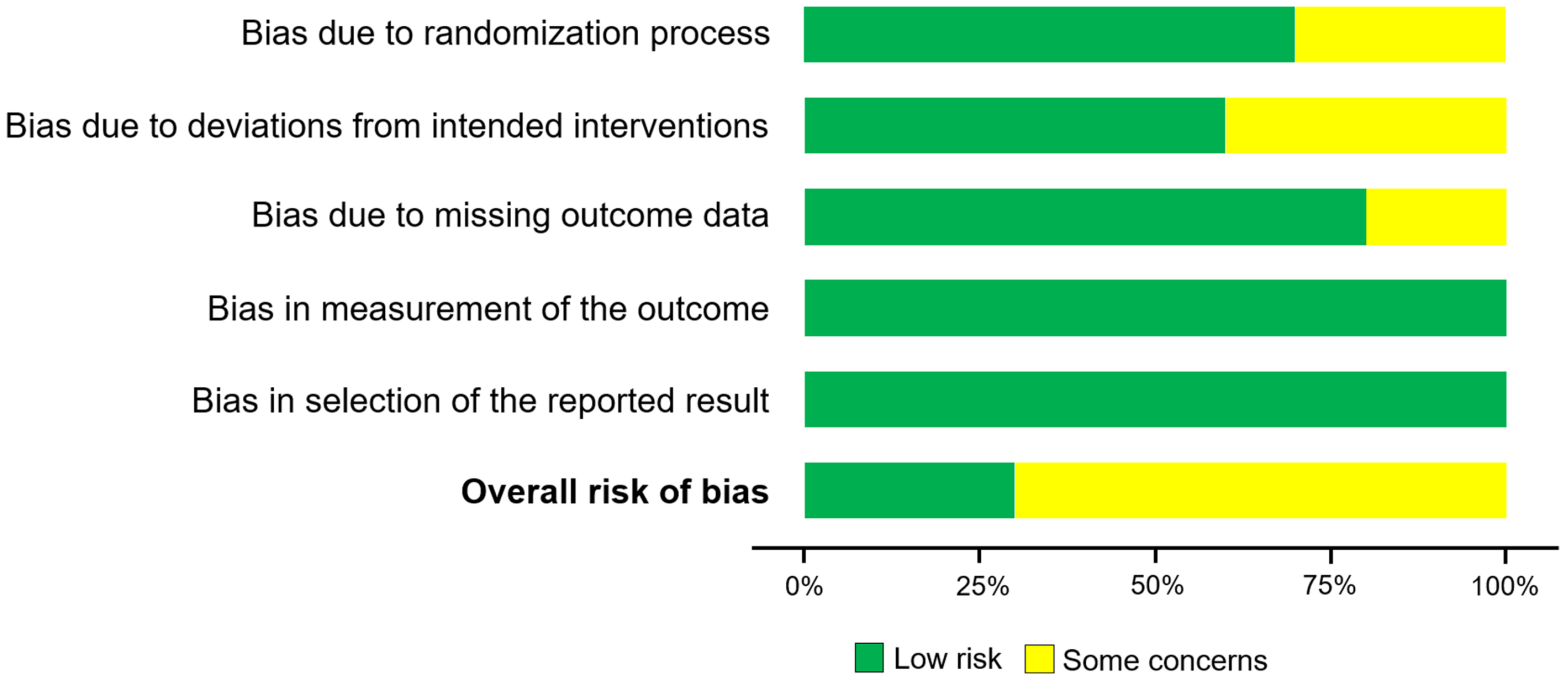
Risk of bias as a percentage. The overall methodological quality was rated as moderate: three studies were judged to be at low risk, while seven were considered to raise some concerns.

**Figure 3:**
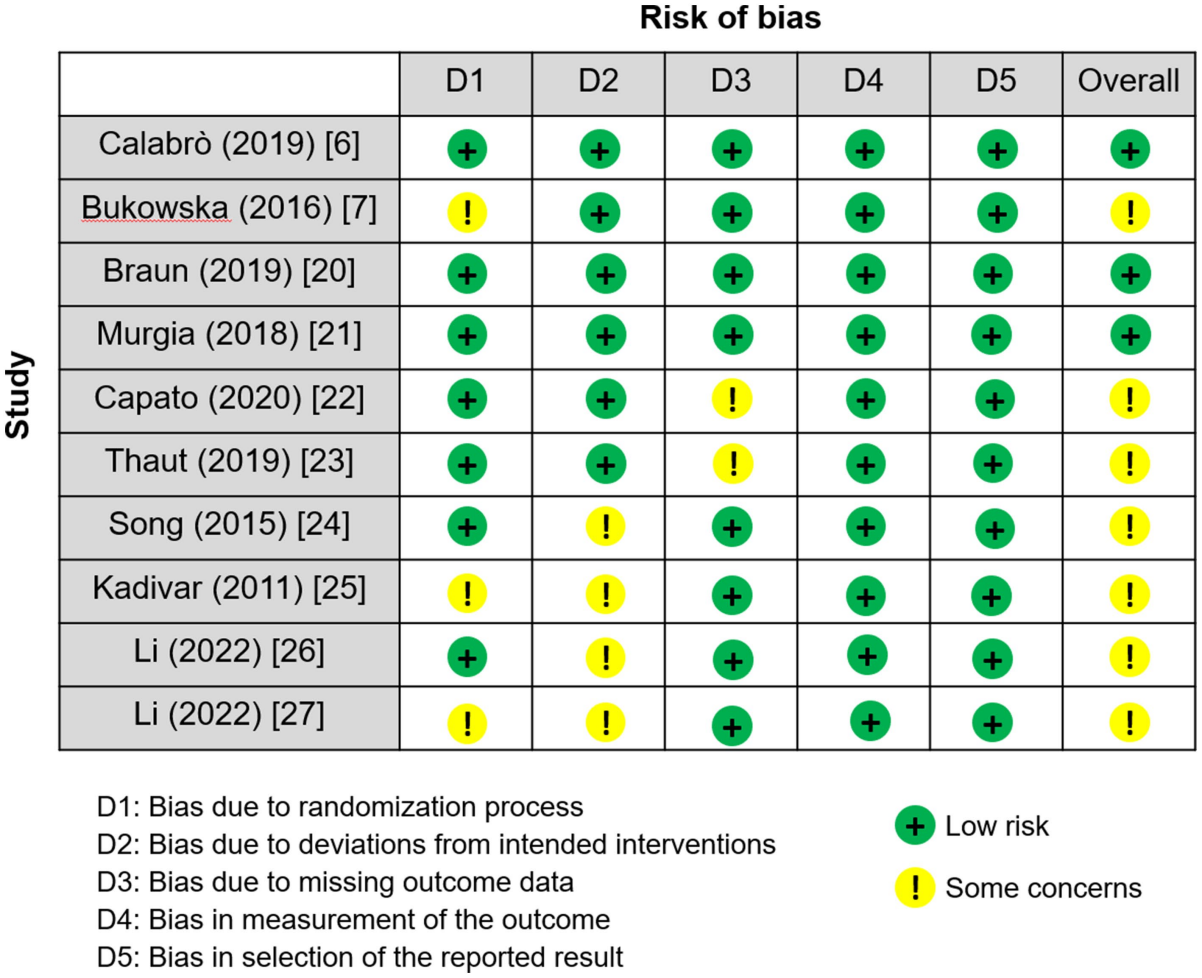
Risk-of-bias summary (6, 7, 20–27). Each study was evaluated across the five domains of RoB 2, with judgments categorized as low risk, some concerns, or high risk.

The overall methodological quality of the included randomized controlled trials (RCTs) was assessed as moderate. Risk of bias outcomes are detailed in Figure 2. Of the 10 studies reviewed, three were rated as “low risk” of bias (6, 20, 21), while the other seven were classified as having “some concerns” (7, 22–27). The most frequent sources of bias were small sample sizes, loss to follow-up, challenges in maintaining full blinding during intervention, and inadequate reporting of the randomization process. Importantly, most studies utilized objective outcome measures and employed either blinded assessments or standardized procedures during evaluation and analysis, which partially reduced bias risk. Accordingly, we adopt a cautious attitude toward the overall certainty of the evidence. Due to the inherent characteristics of music interventions, it is frequently difficult to fully blind both participants and therapists. In some studies, outcome assessors were not blinded, or blinding procedures were insufficiently reported, constituting a further source of bias (28). This limitation is common across behavioral intervention research. Overall, the included studies exhibited moderate methodological quality and risk of bias. See Figures 2, 3 for detailed results. Inter-rater agreement under the RoB 2 criteria was as follows, weighted *κ* values for domain-level judgments ranged from 0.66 to 0.77 (95% CI, 0.50–0.86), and the weighted κ for overall judgments was 0.74 (95% CI, 0.58–0.87). According to the commonly cited thresholds by Landis and Koch, this level of agreement can be considered “substantial.”

### Characteristics of included studies

This review and meta-analysis incorporated 10 randomized controlled trials (RCTs) published from 2011 to 2022, with a combined total of 529 participants. Individual study sample sizes ranged from 16 to 116, with the majority enrolling between 30 and 60 participants. The principal characteristics of these studies are summarized in Table 3 and described in detail below. The results of the PRISMA checklist assessment are shown in Table 2.

1) **Study Population:** All included studies enrolled older adults diagnosed with primary (idiopathic) Parkinson’s disease, with mean participant ages ranging from 63 to 77 years. The sex distribution was generally balanced, although a slight male predominance was observed in some studies; most reported male-to-female ratios between 1:1 and 1.2:1, consistent with the epidemiological profile of Parkinson’s disease. All diagnoses were based on established clinical criteria. Disease severity was primarily mild to moderate or moderate to advanced, with most participants classified within Hoehn & Yahr stages II to IV. The duration of disease typically ranged from 4 to 12 years, although a few studies included patients with more prolonged disease courses.
2) **Intervention (NMT Techniques):** All included studies were conducted within the theoretical framework of NMT and applied structured music-based interventions. The central approach was rhythmic auditory stimulation (RAS) (6, 7, 20–27), commonly used to improve gait, balance, and multidirectional stepping. External temporal cues were typically delivered via metronomes, rhythmic music, step sounds, or customized musical tracks. Training tempo was usually individualized, set according to each participant’s baseline cadence and adjusted upward by 10–20% as appropriate (6, 20, 21, 23–25). In some studies, interventions featured folk songs, classical music, or traditional Chinese music (e.g., yangge dance) (23, 27), and in others, lyric-free rhythmic tracks were tailored to participant preferences to increase engagement (26). Training modalities were varied, including synchronized walking, treadmill training, and multidirectional stepping exercises (6, 23–25). Several studies also examined the immediate transfer effects of rhythmic synchronization of upper limbs (such as finger tapping or arm swinging) on gait performance (20). In addition, RAS was often combined with physical training elements, such as multimodal balance exercises, standard rehabilitation, strength training, or traditional Chinese dance (7, 22, 24, 27). Overall, all interventions emphasized external rhythmic cues and combined personalized music, gait, and multidirectional movement training, with some protocols incorporating multimodal (auditory, visual, and motor) stimulation to systematically improve gait, balance, and functional mobility in individuals with Parkinson’s disease.
3) **Duration and Intensity of Intervention:** Most studies applied intervention durations of 4 to 8 weeks, with each training session typically lasting 30 to 60 min and occurring either three to five times per week or once daily. Protocols frequently prioritized high frequency and sufficient training volume, such as five 30-min sessions per week (6, 24, 26) or four 45-min sessions per week (7); in some cases, intensive regimens included five 60-min sessions weekly (27). One study implemented a long-term strategy, delivering daily sessions over 24 weeks (23) to promote sustained engagement and support ongoing rehabilitation gains. Regarding intensity, most studies gradually increased either the training tempo or task complexity relative to each participant’s baseline cadence—for example, incrementally raising the tempo of the music or metronome, or incorporating multidirectional and more challenging movement tasks (6, 25).
4) **Control Measures:** Control group designs varied considerably across studies. Common comparators included conventional physical therapy, standard rehabilitation protocols, or pharmacological treatments—such as routine walking exercises, strength training, and standard medication regimens—all devoid of musical elements (6, 7, 22, 24–27). Several studies implemented active control groups, where participants engaged in an equivalent amount of physical exercise as the intervention group, but without rhythmic cues, allowing for the specific effects of music or rhythm to be isolated (6, 25–27). Some trials used passive controls, such as medication-only regimens or rest without intervention (7, 20, 24), while others employed music-specific controls, for instance, by comparing different types of rhythmic stimulation or lyric-free music (21, 26). Notably, no studies utilized a pharmacological placebo as a control. In most studies, control protocols were designed to balance therapist attention, training intensity, and intervention duration between groups, thereby minimizing potential attention bias in outcome assessment (7, 25, 26).
5) **Outcome Measures:** All included studies designated motor outcomes as their primary endpoints, with particular emphasis on gait parameters—namely, walking speed, stride length, and cadence. Most studies measured gait velocity, step length, and cadence as common outcome measures. Most studies also examined balance (90%), commonly utilizing instruments such as the BBS, Mini-BESTest, and Tinetti, as well as overall motor function (100%), typically measured by the UPDRS-III and TUG. About half of the studies (50%) further investigated specific motor symptoms, including freezing of gait (FOGQ) (21, 22, 25, 26) and risk of falls (FES-I) (21, 22, 25). Non-motor outcomes were explored in only half of the studies, mainly addressing activities of daily living (UPDRS-II, FIM), while 30% assessed mood or quality of life (FES-I, GDS, PDQ-8). Notably, none of the studies specifically evaluated cognitive function. In addition, 30% incorporated novel mechanistic measures, such as EEG-based neural connectivity (6) and gait kinematic variables (e.g., ankle dorsiflexion, gait cycle phase) (7, 23, 26), thus providing physiological insights into intervention mechanisms.

**Table 3:** Key study characteristics.

First Author (Year)	Country	Sample		Age	NMT intervention (technique; frequency; duration)	Control	Outcomes measured
		Ex	Co				
Calabrò (2019) (6)	Italy	25	25	71.5 ± 8	RAS + treadmill training; 30 min per day, 5 days per week; 8 weeks	Conventional multimodal rehabilitation combined with an equivalent amount of treadmill training (non-RAS)	FGA, FES, UPDRS; GQI; 10MWT; BBS; TUG
Bukowska (2016) (7)	Poland	30	25	63.4 ± 9.6	Multimodal NMT (RAS + PSE + TIMP); 45 min per session, four times per week; 4 weeks	Control group engaging in routine daily activities	Stance phase, swing phase, double-support time, stride time; cadence; step length, stride length, gait speed, etc.
Braun (2019) (20)	Canada, United States	25	12	66.6 ± 4.9	Single-session immediate RAS training; 1 min per set, 3 sets per session; Single-session training	Passive control	Gait velocity; cadence; stride length
Murgia (2018) (21)	Italy	16	16	68.2 ± 10.5	Internal comparison of RAS (footstep sounds vs. metronome); 45 min per session, twice per week; 5 weeks	Both groups received RAS-based gait training; the only difference was the type of auditory stimulus used	Gait speed; cadence; step length; step width; stride length; stance phase, swing phase, double-support phase, etc.
Capato (2020) (22)	Netherlands, Brazil	17	18	77.5 ± 8.5	RAS—supported multimodal balance training; 45 min per session, twice per week; 5 weeks	Conventional rehabilitation control without rhythmic auditory stimulation	Mini-BESTest; BBS; TUG; NFOG-Q; FES-I
Thaut (2019) (23)	Canada, United States	25	22	72 ± 7.5	Home-based daily RAS training; 30 min per session, once daily; 24 weeks	Randomized withdrawal design (treatment-withdrawal-re-treatment)	Fall Index; gait-related dynamic parameters; BBS; TUG; FES
Song (2015) (24)	China	56	56	66.1 ± 7.9	RAS + rhythmic visual stimulation (audio-visual combined); 30 min per session, five times per week; 8 weeks	Conventional pharmacological treatment for Parkinson’s disease	Stride; cadence; gait speed; UPDRS-II; UPDRS-III; BBS; 6MWT
Kadivar (2011) (25)	United States	8	8	70.5 ± 2.2	RAS + step/gait training; 45–60 min per session, three times per week; 6 weeks	Control design using conventional active gait training	DGI; UPDRS; Tinetti Balance and Gait Assessment; TUG; FOGQ
Li (2022) (26)	China	46	24	63.7 ± 10.2	Short-term clinical RAS; 30 min per session, 5 times per week; 4 weeks	Control group receiving standard drug therapy, physical agents, and daily living skills training	MoCA; UPDRS-III, UPDRS-II; FOG-Q
Li (2022) (27)	China	17	34	67.9 ± 6.5	RAS combined with Yangge dance; 60 min per session, 5 times per week; 4 weeks	Conventional exercise group receiving no background music or rhythmic cues	UPDRS; BBS; TUG; Purdue Pegboard Test

Co, control group; Ex, experimental group; RAS, Rhythmic auditory stimulation; FGA, Functional Gait Assessment; FES, Falls Efficacy Scale; UPDRS, Unified Parkinson’s Disease Rating Scale; GQI, Gait Quality Index; 10MWT, 10-Meter Walk Test; BBS, Berg Balance Scale; TUG, Timed Up and Go; Mini-BESTest, Mini-Balance Evaluation Systems Test; FOGQ, Freezing of Gait Questionnaire; DGI, Dynamic Gait Index; MoCA, Montreal Cognitive Assessment.

### Effects of the interventions on motor outcomes

#### Cadence

Based on the available randomized controlled trials (RCTs), we found in some cases that rhythmic auditory stimulation (RAS) may contribute to improvements in cadence. This could mean an increase for patients with low baseline cadence, or, conversely, a decrease for those with abnormally high cadence, leading to a more normalized rhythm. Nevertheless, the pooled meta-analytic results indicated that the overall effect did not reach statistical significance and was accompanied by rather high heterogeneity (SMD = 0.14, *p* = 0.65; I^2^ = 82.6%). Several high-quality randomized controlled trials (RCTs) (7, 20, 21, 23) have shown that NMT interventions can significantly enhance cadence or effectively normalize abnormal cadence. For instance, one study (20) paired finger tapping with RAS, using a metronome set at 20% faster than the participant’s usual walking cadence to guide dominant hand training. This approach increased cadence from 109.25 to 117.5 steps per minute, representing an approximate 8% improvement. Another trial (7) employed a 45-min combined intervention involving RAS, Patterned Sensory Enhancement (PSE), and Therapeutic Instrumental Music Performance (TIMP), with percussion instruments, a metronome, and rhythmic music, resulting in an increase in cadence from 110.38 to 116.64 steps per minute—a statistically significant change (*p* < 0.01). Notably, changes in cadence are not invariably upward. Some studies have observed reductions in cadence with RAS in participants exhibiting festination or excessively high cadence (6, 26). In these instances, NMT may decrease festination and increase stride length, facilitating a transition from abnormally rapid to more physiologically appropriate gait rhythms and resulting in smoother locomotion. Therefore, reductions in cadence in this context reflect clinical improvement, characterized by less festination and a more normalized gait pattern.

Emerging evidence suggests that individualized tempo adjustment, combined with strength training and sustained intervention, plays a critical role in optimizing the efficacy of NMT. Multiple studies (6, 21, 22, 25, 26) indicate that adapting the musical tempo to a patient’s baseline gait speed allows cadence training to better match natural physiological rhythms, resulting in significant reductions in Freezing of Gait Questionnaire (FOGQ) scores and substantial improvements in abnormal cadence and festination. For instance, in an ecological RAS group, cadence increased from 115.81 to 123.40 steps per minute, with gains maintained at a three-month follow-up (21). Similarly, finger-tapping exercises paired with a metronome set 20% above baseline cadence yielded an approximate 8% increase in cadence (20). The use of individualized tempos not only enhances the safety but also the effectiveness of cadence training, underscoring the therapeutic advantages of NMT in motor rehabilitation. Additional studies (7, 27) have shown that integrating NMT with strength training can significantly elevate cadence or normalize it from pathologically high levels, with marked improvements in bradykinesia and freezing of gait. Long-term follow-up evidence is also robust: five studies (6, 21–23, 25) reported that, following several weeks to months of intervention, improvements in cadence or cadence correction persisted for at least 3 months. These benefits were relatively stable throughout the intervention, tended to decline after cessation, but could be restored upon resumption of therapy.

**Figure 4:**
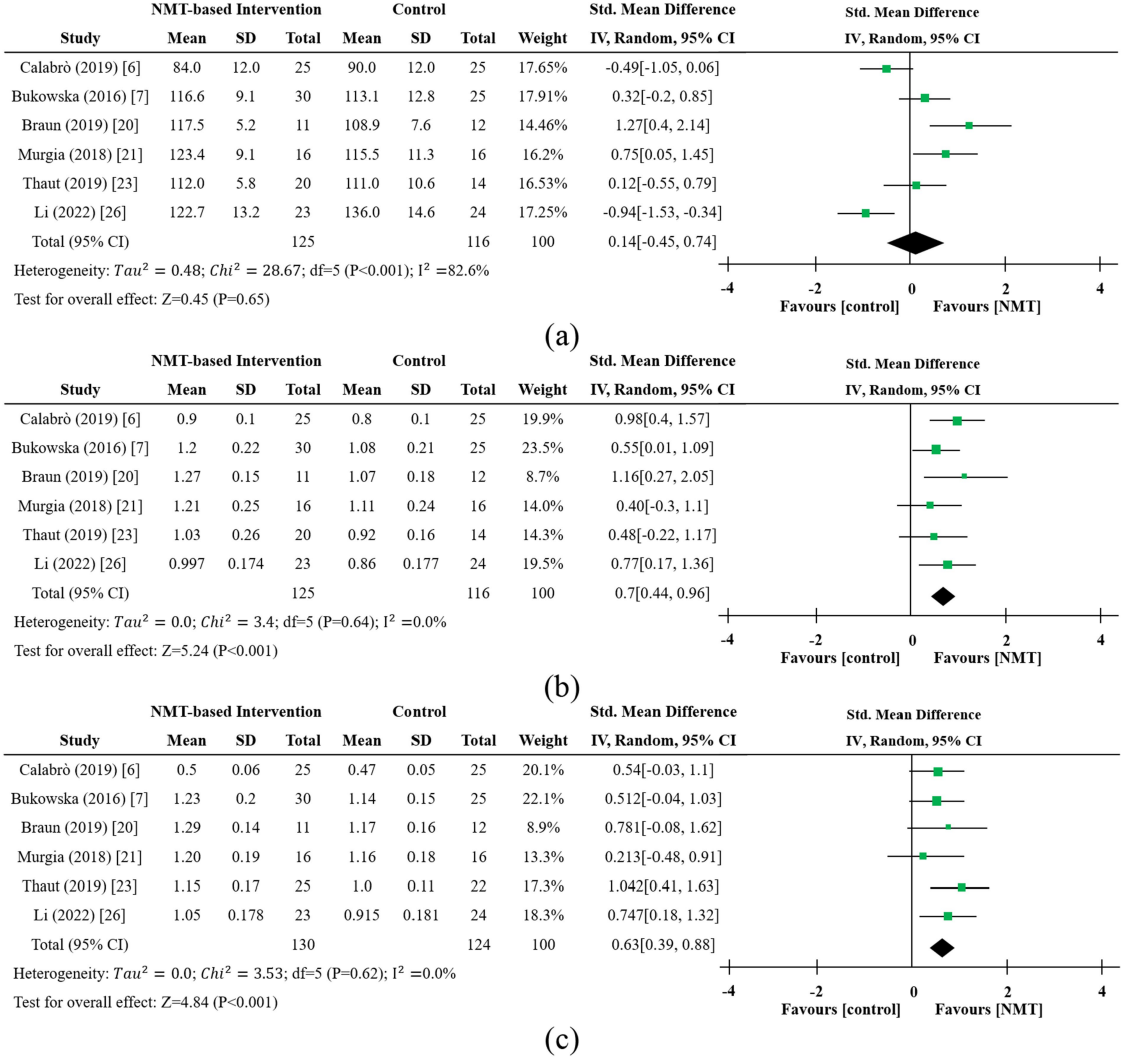
Forest plot for **(a)** cadence (6, 7, 20, 21, 23, 26); **(b)** gait speed (6, 7, 20, 21, 23, 26); **(c)** stride length (6, 7, 20, 21, 23, 26) (RAS-based NMT trials). All pooled effects in were derived from RAS protocols, findings should not be generalized to non-RAS NMT techniques. The size of each square reflects the study weight (inverse variance weighted); the horizontal line indicates the 95% CI, and the diamond represents the pooled effect with its 95% CI.

A total of six studies involving 241 participants examined the impact of NMT on gait cadence in individuals with Parkinson’s disease (6, 7, 20, 21, 23, 26). As shown in Figure 4a, the forest plot displays the mean difference in cadence and corresponding 95% confidence intervals, based on a random-effects model. The overall standardized mean difference (SMD) was 0.14, indicating that NMT may offer a modest improvement in cadence for patients undergoing rehabilitation, though this finding did not reach statistical significance (Z = 0.45, *p* = 0.65). Two studies reported negative effect sizes, where the intervention group performed less favorably than controls, which diluted the combined effect. The I^2^ statistic was 82.6%, reflecting considerable heterogeneity across studies (*p* < 0.001). Sensitivity analysis demonstrated that, after removing two studies (6, 26), heterogeneity was reduced to a moderate level (I^2^ = 39.0%), and the difference between groups became statistically significant (SMD = 0.57, 95% CI [0.282, 0.858], *p* < 0.001). It should be emphasized that some trials (6, 26) explicitly defined the reduction of abnormally high cadence as a therapeutic goal. Accordingly, in the sensitivity analysis, once these two studies (6, 26) were excluded, the pooled heterogeneity dropped markedly from 82.6 to 39.0%, and the effect size shifted from non-significant to statistically significant (SMD ≈ 0.57). However, under our main analytic approach, where a uniform rule of “cadence increase = improvement” was applied, such clinical benefits were paradoxically coded as negative effects, which in turn amplified the heterogeneity driven by differences in effect direction.

#### Gait speed

Most studies included in this systematic review consistently demonstrate that RAS produces significant improvements in both gait speed and stride length among older adults with Parkinson’s disease. Regardless of the specific approach—whether rhythmic auditory stimulation (RAS), footstep cues, or metronome beats—NMT interventions have been shown to effectively accelerate walking speed. Multiple studies (6, 7, 20, 21, 23, 26) reported statistically significant gains in gait speed, with improvements ranging from 9 to 41%. Increased gait speed was closely linked to enhanced motor agility and helped to relieve core symptoms such as bradykinesia and gait freezing. These benefits were reflected not only in the absolute values of gait speed, but also in improved performance on motor function scales and greater daily walking ability. For example, reduced times on the Timed Up and Go (TUG) test indicated faster walking speed and improved agility, while increases in 6-Minute Walk Test (6MWT) distance suggested better endurance and walking efficiency (7, 23). The TUG, a standard measure of functional mobility in older adults, holistically assesses balance and stability across standing, walking, turning, and sitting transitions (29, 30). Additionally, improvements in UPDRS-III, Webster score, and BBS further confirm the functional impact of increased gait speed.

Six studies, comprising a total of 241 participants (6, 7, 20, 21, 23, 26), evaluated the effects of NMT on gait speed. Figure 4b displays a forest plot illustrating the mean difference in gait speed and its 95% confidence intervals, calculated using a random-effects model. The aggregated standardized mean difference (SMD) was 0.70, reflecting a moderate beneficial impact of NMT on gait speed. Notably, the Z-test (Z = 5.24, *p* < 0.001) confirmed the statistical significance of this effect. Two studies (6, 20) demonstrated strong positive outcomes (SMDs of 0.98 and 1.16), whereas one study (21) reported a smaller effect size (SMD = 0.4). The I^2^ statistic was 0% (*p* = 0.64), indicating negligible heterogeneity and high consistency in effect sizes across the included studies.

#### Stride length

Multiple studies included in this systematic review (6, 7, 21, 23, 26) utilized gait analysis systems to capture precise measurements of stride length, offering robust evidence that RAS can substantially increase stride length, mitigate shuffling gait and step length reduction, and promote both the fluidity and normalization of gait in individuals with Parkinson’s disease. Furthermore, some studies (6, 23, 24) inferred stride length improvements indirectly, as indicated by lower Freezing of Gait Questionnaire (FOGQ) scores, longer distances in the 6-Minute Walk Test (6MWT), shorter Timed Up and Go Test (TUGT) times, and reduced UPDRS-III or Webster scores.

Six studies, encompassing a total of 254 participants (6, 7, 20, 21, 23, 26), evaluated the impact of NMT on stride length. As illustrated in Figure 4c, the forest plot displays the mean differences and 95% confidence intervals calculated with a random-effects model. The meta-analysis demonstrated a statistically significant enhancement in stride length following NMT (SMD = 0.63, 95% CI = 0.39–0.88, *p* < 0.001), with negligible heterogeneity among studies (I^2^ = 0%). Overall, these results indicate that NMT may effectively improve stride length in individuals with Parkinson’s disease.

#### Other motor functions

While gait improvement remains the central focus of most research, the majority of studies have also provided systematic assessments of additional motor outcomes:

1) **Balance and Postural Control:** Across 6 studies, NMT has been shown to significantly improve balance in individuals with Parkinson’s disease. Evidence from diverse assessment instruments—including the Berg Balance Scale (BBS) (6, 23, 27), Tinetti Scale (21, 25), and Mini-BESTest (22)—consistently demonstrates that participants receiving NMT exhibit greater gains in balance compared to control groups. Notably, one study (7) found that the benefits of NMT for postural stability were especially evident under demanding conditions, such as eyes-closed testing or sensory challenges, highlighting the therapy’s potential to enhance proprioception and multisystem integration. Moreover, related studies indicate that NMT can markedly reduce fear of falling, decrease the actual incidence of falls, and promote greater confidence and engagement in physical activity.

**Figure 5:**
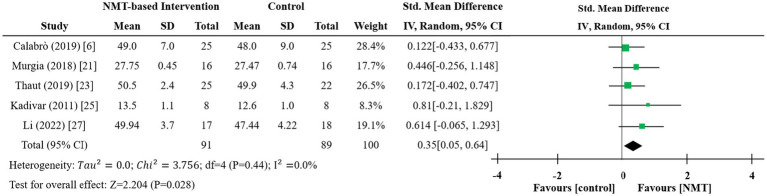
Forest plot for balance and postural control (6, 21, 23, 25, 27). The size of each square reflects the study weight (inverse variance weighted); the horizontal line indicates the 95% CI, and the diamond represents the pooled effect with its 95% CI.

Among the six studies reviewed, one lacked complete descriptive statistics (22), so the meta-analysis was restricted to the remaining five studies (6, 21, 23, 25, 27). Marked differences in means, standard deviations, and sample sizes, together with the use of different assessment instruments (including the Berg Balance Scale and Tinetti Balance Scale), resulted in notable clinical and methodological heterogeneity. Consequently, a random-effects model was deemed most appropriate. Figure 5 presents the forest plot illustrating mean differences in balance scores and their corresponding 95% confidence intervals. The pooled standardized mean difference (SMD) was 0.35 (Z = 2.204, *p* = 0.028), indicating a statistically significant overall effect at the 5% significance level under the random-effects model. This finding suggests that NMT may provide a small to moderate benefit in improving balance. Both Tau^2^ = 0 and I^2^ = 0% demonstrate minimal heterogeneity among studies, further supported by a Q-test *p* value of 0.44. Given the broad range of mean values (13.5 to 50.5) and the small sample size of one study, a sensitivity analysis was conducted. After excluding study (27), heterogeneity remained negligible (I^2^ = 0.0%), but the effect was no longer statistically significant (SMD = 0.289, 95% CI [−0.04, 0.618], *p* = 0.085), as the p value exceeded 0.05 and the confidence interval included zero.

2) **Lower Limb Function and Overall Motor Capacity:** Some studies have also examined changes in endurance, agility, and functional strength of the lower limbs. For example, one study assessed lower limb endurance using the 6-Minute Walk Test (6MWT), finding that after 8 weeks of combined rhythmic auditory and visual cue training, the distance covered in the 6MWT increased significantly (24). However, the effectiveness and sustainability of NMT on Timed Up and Go (TUG) performance in older adults with Parkinson’s disease varies across studies. Several randomized controlled trials have reported that NMT can significantly reduce TUG time, thereby improving motor coordination and walking safety. For instance, reference [6] reported that 8 weeks of treadmill training combined with RAS reduced TUG time from 11 s to 9 s (a 22% improvement, *p* < 0.001), whereas the conventional training group improved from 11 s to 10 s (a 10% improvement, *p* = 0.01); another study combining multidirectional gait training with RAS found that TUG times for some patients dropped below 7.95 s, with benefits lasting at least 8 weeks (25). Additionally, another controlled trial (27) demonstrated that rhythmic cues (such as yangge dance or music-assisted exercise) produced greater improvements in TUG than conventional exercise. However, improvements in TUG are relatively limited among patients with advanced Parkinson’s disease. Reference [22] indicated that Hoehn & Yahr stage patients experienced a mean reduction in TUG time from 29.8 s to 23.6 s after RAS training, but the control group showed no significant change, and the between-group difference did not reach statistical significance (*p* = 0.065). Similarly, reference [23] reported no significant improvement in TUG after home-based RAS training among high-fall-risk patients, suggesting that the effects of standalone music interventions may be constrained by baseline motor capacity and cognitive status in late-stage disease or functionally limited populations.

#### Strength of evidence for motor outcomes

In summary, this review draws on multiple high-quality studies that consistently show NMT, particularly interventions based on rhythmic auditory stimulation, can bring about significant improvements across diverse motor domains in individuals with Parkinson’s disease. Most randomized controlled trials and prospective studies utilized blinded designs, quantitative assessments, and multidimensional outcome measures, supporting findings with robust statistical power and strong clinical relevance. Moreover, combined training protocols (such as multidirectional gait training, integrating music with exercise, or pairing rhythmic cues with visual prompts) appear more effective than rhythmic auditory stimulation alone. Enhancements in functional scales, such as UPDRS-III, TUG, BBS, and DGI, further substantiate the capacity of NMT to alleviate motor deficits and foster greater independence in daily living. Notably, several studies report that these motor gains are well maintained, with many patients preserving their improvements for months after intervention, alongside reduced fall rates, less freezing of gait, and enhanced confidence in rehabilitation.

Analysis of the available evidence suggests that NMT is supported by moderate- to high-quality clinical data for enhancing motor outcomes in older adults with Parkinson’s disease. For motor outcomes, the pooled effect sizes for gait speed and stride length were both in the moderate range, with negligible heterogeneity, making these conclusions relatively robust. By contrast, the pooled effect for cadence did not reach statistical significance and was accompanied by high heterogeneity. Although sensitivity analyses suggested some potential signals, these findings can only be regarded as exploratory. As for balance, the results proved extremely sensitive to the exclusion of individual studies, suggesting that the overall evidence is weak and of limited reliability.

### Effects of the intervention on non-motor outcomes

Beyond motor outcomes, this review also highlights studies exploring the potential impact of Neurologic Music Therapy (NMT) on non-motor symptoms in Parkinson’s disease (PD). While the evidence for these effects is comparatively limited and variable, preliminary findings offer valuable insights.

#### Quality of life

Currently, only a small number of studies have offered direct evidence that NMT can improve quality of life (QoL) in individuals with Parkinson’s disease. For example, one study (21) employing the PDQ-8 QoL scale found that, at 3 months post-intervention, participants experienced significant improvements in QoL compared to baseline (*p* < 0.01), regardless of whether ecological footstep sounds or metronome-based rhythmic auditory stimulation was used. This finding provides direct and robust clinical support for the efficacy of NMT in enhancing QoL. Other studies have inferred possible benefits of NMT for QoL based on observed improvements in motor function, activities of daily living, and social engagement; however, the evidentiary strength here is constrained by the lack of direct quantitative assessment. For instance, some reports have linked gains in mobility and ADLs with improved QoL (7), while others have suggested that enhanced manual dexterity and self-care may also be beneficial (27). Additionally, one study (26) lacking a standardized QoL scale provided indirect support for NMT’s impact through improvements in freezing of gait (FOG) and motor function, as indicated by reductions in FOG-Q scores. Only one RCT employed PDQ-8 to directly quantify QoL, indicating improvement up to 3 months; however, most other reports infer QoL benefits indirectly from motor or ADL gains, representing low-level evidence that does not support firm conclusions.

#### Emotional well-being

Only a single study has employed the Geriatric Depression Scale (GDS) (21) to longitudinally evaluate emotional changes in individuals with Parkinson’s disease after receiving ecological or artificial rhythmic auditory stimulation. This study reported a downward trend in GDS scores following 5 weeks of intervention and at three-month follow-up, suggesting a possible reduction in depressive symptoms. However, this change did not achieve statistical significance after adjustment for multiple comparisons, indicating limited strength of evidence. Most other included studies (6, 7, 20, 22–25) relied on prior research or theoretical frameworks in their discussions, positing that NMT may support emotional well-being by enhancing motor performance, increasing participation and social interaction, promoting positive emotions, and improving motivation and treatment adherence, though these effects were not empirically measured in the studies themselves. Moreover, while most research did not formally quantify emotional health outcomes, qualitative and anecdotal observations commonly described increased positivity and self-confidence among participants undergoing music interventions. Engagement in musical activities has also been considered potentially beneficial for alleviating apathy, a frequently observed symptom in Parkinson’s disease, although, to date, no direct quantitative evidence substantiates this assumption.

#### Cognitive function

The majority of studies included in this systematic review did not identify cognitive function as a primary outcome, resulting in a notable scarcity of quantitative clinical evidence in this domain. In one study (24), cognitive screening was incorporated into the inclusion criteria by requiring participants to achieve an MMSE score of 28 or higher to exclude overt dementia. However, the intervention period did not involve systematic assessments of cognitive flexibility, attention, or executive function, nor were cognitive follow-up tasks administered. Another study (6) employed electroencephalography (EEG) and observed that rhythmic auditory stimulation increased alpha connectivity between the prefrontal and parietal cortices, implying a possible role for such interventions in modulating motor-cognitive rhythmic networks and suggesting indirect cognitive benefits of NMT. Nevertheless, this study did not employ specific cognitive behavioral tasks or standardized cognitive scales, so its conclusions remain limited to neurophysiological observations without direct behavioral corroboration. Overall, clinical evidence for NMT’s cognitive effects is still in its nascent stages, and definitive conclusions cannot yet be drawn. Some reports indicate that improvements in cognitive function may emerge when interventions explicitly target cognitive engagement, such as through rhythmic cognitive games or music-based multitasking exercises. This approach is consistent with the theoretical underpinnings of cognitive training modules in NMT (e.g., musical attention control training), but such protocols remain underrepresented in current randomized controlled trials for Parkinson’s disease.

#### Other non-motor outcomes

This systematic review revealed that most included studies did not systematically assess non-motor outcomes such as speech and voice, inhibitory control, executive function, or social functioning. These domains were typically referenced only in mechanistic discussions and remain underexplored from an empirical perspective. With respect to activities of daily living (ADL), several studies (21, 22, 25) employed the UPDRS-II (Activities of Daily Living subscale) and FIM (Functional Independence Measure) as secondary outcome measures. However, the majority of these studies observed no significant post-intervention improvements in ADL scores, or the differences between groups did not reach statistical significance, indicating that direct quantitative evidence supporting the efficacy of NMT in enhancing ADL remains limited. A number of studies evaluated patients’ fear of falling and movement confidence using measures such as the FES-I (Falls Efficacy Scale-International), Tinetti Balance Scale, and ABC (Activities-specific Balance Confidence Scale). Five studies (6, 7, 21–23) reported improvements in fear of falling or confidence scores in the NMT group, and in some cases (6, 21, 23) these effects persisted through follow-up. Nonetheless, these findings were largely reported as secondary outcomes and did not always achieve statistical significance between groups. Overall, NMT appears to support reductions in fear of falling and improvements in movement confidence for some patients, particularly when integrated with gait or balance training. Regarding proprioception, one study (7) found that NMT, incorporating RAS, TIMP, and PSE, significantly enhanced balance under eyes-closed conditions, suggesting potential benefits for proprioceptive and postural awareness in individuals with Parkinson’s disease. Conversely, improvements in static stability with eyes open were more modest. These results imply that music- and rhythm-based interventions may primarily facilitate intrinsic sensory-motor regulation, compensating for deficits in postural control when visual feedback is reduced.

#### Strength of evidence for non-motor outcomes

This systematic review demonstrates that direct evidence supporting the effects of NMT on non-motor outcomes in Parkinson’s disease remains scarce. Most rigorous clinical trials have not implemented systematic or standardized quantitative assessments for non-motor domains. Currently, quality of life stands out as one of the few non-motor outcomes for which direct evidence exists. A single randomized controlled trial (RCT) using the PDQ scale found that NMT led to significant improvements in quality of life, with benefits maintained for up to 3 months post-intervention, providing moderate-to-strong support for this outcome. In contrast, most other studies did not employ formal quality of life metrics, instead inferring potential benefits indirectly from improvements in motor function or activities of daily living (ADL), which weakens the overall evidence base. With respect to emotional health, only a limited number of studies have observed a downward trend in depressive symptoms (e.g., GDS scores) following NMT interventions; however, these changes generally did not achieve statistical significance, and there have been no systematic assessments of anxiety or other emotional domains. The majority of studies either omitted validated emotional health measures or addressed the potential mood-modulating effects of NMT in speculative discussions, resulting in a limited and inconclusive evidence base.

Similarly, no studies to date have systematically and quantitatively assessed cognitive function as a primary or secondary outcome. Some research has relied on neurophysiological approaches, such as EEG, to propose that NMT may influence motor-cognitive networks; yet these observations are based on neural mechanisms and lack corroborating behavioral or scale-based data, thus remaining theoretical. Other non-motor outcomes, including speech and voice, social functioning, ADL, fear of falling/self-confidence, and proprioception, have seldom been investigated using direct quantitative methods. Only a small number of studies have followed up on ADL or fall-related confidence with validated scales, and most reported improvements did not reach statistical significance, limiting the ability to draw firm conclusions. For outcomes such as speech, inhibitory control, and social participation, current evidence is restricted to mechanistic speculation without quantitative evaluation.

In summary, while NMT shows promise for enhancing quality of life and psychosocial wellbeing in Parkinson’s disease, the strongest existing evidence is still concentrated in the domain of motor symptoms. Robust, systematic clinical studies are needed to clarify the benefits of NMT for non-motor symptoms.

## Discussion

### Main findings

This systematic review and meta-analysis synthesizes evidence from randomized controlled trials (RCTs) published between 2011 and 2022 examining the effects of Neurologic Music Therapy (NMT) on rehabilitation outcomes in older adults with Parkinson’s disease (PD). The review team implemented stringent selection and quality appraisal processes, providing a comprehensive summary of NMT’s effects on both motor and non-motor symptoms in this population. Unlike earlier reviews, this study integrates recent English-language research and critically appraises emerging developments in the field. Our findings suggest that NMT, particularly RAS, exerts moderate effects in improving gait speed and stride length, with statistically significant differences and high consistency across studies. By contrast, cadence did not reach significance in the main analysis and was accompanied by high heterogeneity; only after sensitivity analyses [excluding (6, 26)] did a moderate effect emerge alongside a reduction in heterogeneity, indicating that this signal is likely driven by study selection and protocol differences and should not be generalized as an overall conclusion. For balance, the pooled effect showed only minor improvements and was highly dependent on the inclusion of specific studies, becoming non-significant once (27) was excluded. Evidence for non-motor outcomes remains limited and inconsistent, relying largely on indirect measures or single-study findings.

At the motor symptom level, a range of primary studies included in this review provide consistent evidence that NMT, and in particular rhythmic auditory stimulation (RAS), facilitates multidimensional improvements in motor function among individuals with Parkinson’s disease (PD). Multiple randomized controlled trials have found that NMT interventions significantly enhance gait speed and stride length, with some also noting concurrent gains in cadence (6, 7, 21, 23, 24). The current literature suggests that rhythmic interventions, especially RAS, effectively optimize spatiotemporal and kinematic gait parameters in PD, leading to better gait velocity, stride length, overall motor performance, and balance (31). Rhythmic musical cues not only accelerate walking speed (with improvements of approximately 9 to 41%) and increase stride length, but also reduce festination and foster a more normalized, stable gait. Both ecological footstep cues and artificial metronome beats have demonstrated sustained benefits for gait outcomes, with improvements maintained for up to 3 months after intervention (21). By serving as external temporal cues, rhythmic auditory signals such as metronome beats or pronounced musical accents precisely regulate gait timing and pacing, potentially compensating for basal ganglia dysfunction and supporting motor rehabilitation through activation of compensatory neural networks (32–35).

Intervention approaches that integrate rhythmic auditory stimulation (RAS) with multidirectional stepping, treadmill-based music training, or visual cueing (6, 24) have been shown to enhance not only the spatiotemporal characteristics of gait in individuals with Parkinson’s disease, but also dynamic balance and postural stability. These benefits are consistently evidenced by improvements in validated clinical assessments, including the Berg Balance Scale, Mini-BESTest, Dynamic Gait Index, and Tinetti Scale. Numerous studies have demonstrated that NMT can meaningfully lower both the risk of falls and the occurrence of gait freezing, while also fostering improvements in gait rhythmicity and motor coordination (21, 23–25). Moreover, participants in the NMT groups achieved significant gains in key functional outcomes such as the motor subsection of the Unified Parkinson’s Disease Rating Scale (UPDRS-III), the Timed Up and Go (TUG) test, and the 6-Minute Walk Test (6MWT), as well as increased independence in daily activities and reduced motor disability.

Meta-analysis demonstrates that RAS-based NMT confers distinct rehabilitative benefits for older adults with Parkinson’s disease. Notably, NMT yields a moderate and statistically significant improvement in gait velocity (SMD = 0.70, 95% CI [0.39, 1.01], *p* < 0.001) and stride length (SMD = 0.63, 95% CI [0.39, 0.88], *p* < 0.001), both with negligible heterogeneity across studies (I^2^ = 0%). Although NMT was associated with a minor positive effect on cadence (SMD = 0.14, 95% CI [−0.46, 0.74], *p* = 0.65), this finding was not statistically significant and exhibited substantial heterogeneity (I^2^ = 82.6%). Sensitivity analysis, after excluding two studies, revealed a significant effect on cadence (SMD = 0.57, 95% CI [0.28, 0.86], *p* < 0.001), with heterogeneity reduced to a moderate level (I^2^ = 39.0%). Regarding balance, NMT may offer a small-to-moderate benefit (SMD = 0.35, 95% CI [0.04, 0.66], *p* = 0.028) with minimal heterogeneity (I^2^ = 0%), although this effect became non-significant after one study was excluded (SMD = 0.29, 95% CI [−0.04, 0.62], *p* = 0.085). In contrast to the consistent and robust improvements in gait speed and stride length, evidence for balance is limited and highly sensitive to sensitivity analysis. Therefore, clinical implications should be cautious, pending confirmation from larger multicenter RCTs.

In addition, research has demonstrated that NMT interventions can markedly improve proprioceptive abilities and balance control when visual input is absent, further substantiating their benefits for both postural and dynamic stability (7). The current body of evidence consistently affirms the safety of NMT, with no serious adverse events reported and high patient adherence observed. Some investigations have examined the use of home-based gait training protocols combined with portable music devices, underscoring the practicality of sustained rehabilitation. Notably, the majority of evidence for motor outcomes is derived from prospective, randomized, blinded trials with moderate to large cohorts, featuring thorough quantitative assessment and robust statistical methods, which collectively underscore the strong clinical promise of NMT.

Among the included studies, the control conditions ranged from active rehabilitation or intensity-matched interventions (e.g., walking training without rhythmic cues or conventional physiotherapy) to usual care or even passive controls. Such variation directly influenced both the pooled effects and the heterogeneity of the primary gait outcomes. When active controls were employed with training duration and intensity closely matched to NMT [e.g., (6), which used multimodal treadmill rehabilitation without RAS as the comparator], the incremental effect of NMT largely reflected the “rhythmic/music” component alone, and pooled effects tended to be more conservative. In contrast, when the comparator was only usual care or daily activity [e.g., (7)], the intervention group not only received rhythmic cues but also benefited from more structured motor–perceptual integration training. With such relatively “weak” controls, nominal effect sizes were often larger, and cross-study pooling could produce systematic bias and uneven weighting, thereby amplifying overall heterogeneity. Overall, the systematic differences in control conditions (active matched vs. usual care) represent a key factor underlying the divergent pooled findings across gait outcomes: gait speed and stride length appeared robust to such design differences (I^2^ ≈ 0%), whereas cadence was highly sensitive to intervention targets and prescription design (initial I^2^ ≈ 83%, which decreased following sensitivity analyses).

Evidence from the included systematic reviews and meta-analyses suggests that while NMT interventions share some structured features in terms of training “dose” and “intensity” the exact protocols differ across studies, and these variations are closely linked to effect size. As shown in Table 3, intervention dosage spanned a gradient ranging from acute, single-session interventions to mid- and long-term follow-up programs (6, 7, 20, 23, 25, 26). With respect to intensity, a common approach was to individualize rhythmic adjustments to each patient’s baseline cadence, gradually increasing tempo or music speed by 10–20% to provide sufficient temporal structure and to challenge sensorimotor coupling (6, 20, 21, 23–25). From the pooled evidence, cadence, being especially sensitive to rhythmic regulation, exhibited a typical “dose—intensity—heterogeneity” interplay: across six studies (*n* = 241), the pooled effect size for cadence was modest (SMD = 0.14), non-significant, and highly heterogeneous (I^2^ = 82.6%), reflecting substantial variability in dosing, intensity, and design (6, 7, 20, 21, 23, 26). However, in sensitivity analyses, excluding two particular studies (6, 26) reduced heterogeneity to a moderate level (I^2^ = 39.0%) and yielded a significant pooled effect (SMD = 0.57, *p* < 0.001). This indicates that distinctive dosing and intensity configurations in some trials may have diluted or exaggerated the overall effect. In summary, the therapeutic impact of NMT should not be seen as a “fixed constant” but rather as jointly shaped by both “dose” (e.g., weekly frequency, session duration, total intervention period) and “intensity” (e.g., tempo increments, progressive task complexity).

Neurophysiological studies indicate that NMT (including RAS, PSE, TIMP, etc.) can regulate rhythmic dynamics across prefrontal, parietal, and motor cortical networks, thereby supporting the neural basis for enhanced motor function observed in clinical settings (6). Deeper exploration into how the brain processes musical cues is instrumental in unraveling the mechanisms of NMT and establishing a solid theoretical foundation for its clinical application (36). Notably, music-induced neural activation can extend beyond musical contexts, promoting improvements in diverse cognitive and motor functions that are clinically quantifiable (37, 38). Owing to its noninvasive profile, NMT is increasingly recognized as an effective complement to traditional cognitive rehabilitation and neuromodulation interventions, highlighting its broad therapeutic promise (39).

Regarding non-motor symptoms, most studies have not undertaken comprehensive quantitative assessments of specific cognitive domains, such as cognitive flexibility or executive function; instead, the existing evidence is predominantly based on overall MoCA scores and lacks domain-specific evaluation or direct evidence. Some studies (21) employing quality of life instruments, such as the PDQ-8, have demonstrated that rhythmic auditory stimulation (RAS) can meaningfully enhance patients’ perceived quality of life, with benefits maintained at 3 months post-intervention. While certain researchers have proposed that improvements in motor function may contribute to better quality of life, such assertions remain largely inferential. Only a handful of studies (21) have observed decreases in GDS depression scores after RAS intervention, though these changes did not achieve statistical significance. Systematic assessment of emotional health is uncommon, with most findings limited to descriptive trends or theoretical speculation. Some reports suggest that improvements in cognitive and motor function may indirectly benefit mood, but quantitative data on outcomes like depression or anxiety remain scarce. Although evidence for emotional health, speech function, and social participation is relatively limited, a small number of studies have investigated NMT for swallowing and speech impairments in Parkinson’s disease, suggesting potential benefit (40). NMT modalities focused on language rehabilitation, including Melodic Intonation Therapy (MIT) and Musical Speech Stimulation (MUSTIM), have received growing attention in recent years (41–43). Compared with motor outcomes, quantitative evidence for non-motor domains is scarce. Direct QoL support is limited to a single RCT; mood outcomes lack consistent, statistically significant scale-based findings; and cognition is largely unquantified. Thus, non-motor effects should be framed as preliminary/limited, not definitive, and prioritized for future trials.

### Potential moderators

This review suggests that several factors may moderate the effects of NMT and, to some extent, contribute to the heterogeneity observed across studies. First, disease stage and baseline risk may determine the “window of plasticity” for intervention. Most participants were patients in Hoehn & Yahr stages II-IV. For example, in a home-based RAS trial, no improvement in TUG was observed among individuals with a high risk of falls, suggesting that in later disease stages or in populations with limited functional reserve, the detectable effects of NMT may be constrained (23). Second, prescription dosage and training intensity varied considerably. Most protocols lasted 4–8 weeks, with 3–5 sessions per week of 30–60 min each, although some high-dose strategies involved daily training over 24 weeks. Typically, rhythmic cues were set relative to each individual’s baseline cadence, with tempo or task complexity progressively increased by 10–20% to provide graded challenges (6, 7, 23–27). These elements of “high-frequency, progressive, and individualized” training qualitatively align with the more consistent positive signals we observed for gait speed and stride length. Third, there were marked differences in control conditions. Some trials employed intensity-matched active rehabilitation as the comparator, others used passive or usual-care controls, and still others applied non-rhythmic music or alternative rhythmic cues. Active, dose-matched controls tended to narrow between-group differences, whereas usual-care comparators were more likely to reveal apparent advantages of NMT (6, 7, 21, 24–27). This variability may help explain the inconsistent pooled effects across different outcomes. In addition, the types of cues and training modes were diverse. Beyond metronomes, studies frequently used rhythmic music, footstep sounds, or individualized non-lyrical tracks. Some protocols also combined RAS with strength training or multimodal balance training, which may have produced synergistic benefits compared with RAS alone (7, 22, 24, 27). Taken together, these interacting factors contributed to the high heterogeneity and instability observed for cadence in the main analysis (SMD = 0.14, I^2^ = 82.6%), with some studies even suggesting negative effects. Future research should aim to standardize subgroup stratification, dosage prescriptions, and control designs, in order to more clearly delineate the true effects of NMT across different patient populations and intervention contexts.

### Clinical significance

The results of this review and meta-analysis underscore the significant clinical value of RAS-based NMT in the rehabilitation of older adults with Parkinson’s disease. Incorporating NMT/RAS into standard rehabilitation protocols has been shown to markedly improve gait, and motor coordination in this population. As a low-cost, user-friendly, and safe adjunctive treatment, NMT reduces the risk and fear of falling. Its flexibility allows for application in a variety of settings, including hospitals, rehabilitation centers, and at home, supporting long-term functional maintenance. Importantly, NMT emphasizes interdisciplinary collaboration and personalized intervention, enabling physical therapists, music therapists, and rehabilitation physicians to collaboratively design tailored programs based on each patient’s abilities and needs. This makes NMT particularly appropriate for high-risk individuals experiencing gait abnormalities, freezing of gait, or cognitive impairment. Current evidence indicates that NMT can increase adherence to rehabilitation and relieve negative emotions such as depression and apathy, thereby enriching the rehabilitation process. Based on the body of evidence included in this review, the overall strength of evidence for NMT/RAS remains at a moderate level, with notable heterogeneity across intervention protocols and outcome measures. While NMT shows relatively consistent improvements in gait speed and stride length, the evidence for cadence, balance, and non-motor outcomes is limited and less stable. Therefore, we position NMT as a promising but cautious adjunctive intervention, best applied within a standardized rehabilitation framework and in combination with conventional therapies after individualized assessment. Its broader adoption will require confirmation through large-sample, multicenter randomized controlled trials with standardized protocols and long-term follow-up.

### Limitations

While this review and meta-analysis systematically evaluates the use of NMT in the rehabilitation of older adults with Parkinson’s disease, several limitations should be noted at both the primary study and review levels. Most included studies were characterized by small sample sizes and short intervention durations, limiting the ability to assess long-term outcomes and the scalability of clinical implementation. Additionally, there was considerable heterogeneity in intervention protocols and outcome assessments, including differences in music selection, frequency and intensity of interventions, and types of outcome measures, which complicated data synthesis and cross-study comparisons. The inherent nature of music-based interventions often precluded rigorous blinding, increasing the susceptibility of subjective outcomes to expectancy effects. Furthermore, robust quantitative evidence for non-motor domains, such as cognitive function, emotional health, and quality of life, remains scarce. Some studies also suffered from incomplete reporting, such as missing follow-up data, unclear reasons for withdrawal, or insufficient documentation of adverse events, all of which impact the completeness and credibility of the findings. Future research should address these gaps by increasing sample sizes, extending follow-up periods, standardizing interventions and outcome measures, improving blinding protocols to strengthen the evidence base for NMT’s clinical adoption and broader implementation. In addition, most of the studies included in this review were rated as “some concerns” by the RoB 2 assessment (e.g., insufficient reporting of randomization, restricted blinding, limited sample sizes), which reduces the certainty of evidence for certain outcomes. Therefore, for conclusions other than gait speed and stride length, we have taken a more cautious interpretive stance, recognizing that evidence quality and external validity still require further confirmation and strengthening in future research. It should be noted that this review did not systematically include Embase, PsycINFO, or Scopus. Although our search strategy combined PubMed, Web of Science, and ProQuest, supplemented by citation tracking and manual searches, this database selection may still introduce coverage bias, particularly with regard to interdisciplinary studies and regional journals. Future research could expand to additional databases to enhance the robustness and representativeness of the findings.

## Conclusion

This review provides a comprehensive evaluation of English-language studies published between 2011 and 2022 on the use of NMT in the rehabilitation of older adults with Parkinson’s disease (PD). The evidence demonstrates that RAS-based NMT can markedly enhance gait speed, stride length in elderly individuals with PD, as well as reduce the risk of falls and help alleviate freezing of gait. With respect to cadence, the current pooled evidence did not show significant effects and was characterized by high heterogeneity. Although some individual studies suggested possible signals of improvement or a trend toward rhythmic normalization, the evidence remains insufficient to support firm conclusions. More rigorous subgrouping and standardized protocols are needed to validate these findings. Future multicenter RCTs should clearly define the target direction, apply consistent control conditions, and systematically report intervention dosage prescriptions in order to further clarify the true magnitude of cadence effects. With respect to balance, the available evidence is both limited and inconsistent. Although a few studies suggested small potential effects, the pooled results proved highly sensitive to the inclusion or exclusion of individual trials, making the conclusions difficult to sustain. Therefore, no definitive inference should be drawn at this stage. Integrating personalized rhythmic cues with multimodal training strategies appears to further strengthen intervention outcomes, with benefits lasting for several months. Thanks to its enjoyable and noninvasive characteristics, NMT also improves patient adherence to rehabilitation and increases subjective satisfaction. While preliminary findings indicate potential benefits of NMT for non-motor outcomes, such as quality of life, cognitive performance, and emotional well-being, the supporting evidence remains limited. This underscores the need for robust, large-scale, long-term randomized controlled trials. Overall, NMT is a safe and cost-effective adjunctive therapy with strong potential to enhance both motor function and quality of life. Ongoing efforts should prioritize standardization and multicenter collaboration to facilitate broader clinical adoption.

## Data Availability

The original contributions presented in the study are included in the article/supplementary material, further inquiries can be directed to the corresponding author.

## References

[1] Thaut MH Hoemberg V. Handbook of neurologic music therapy. New York, NY: Oxford University Press (2014).

[2] Postuma RB Aarsland D Barone P Burn DJ Hawkes CH Oertel W . Identifying prodromal Parkinson's disease. Pre-motor disorders in Parkinson's disease. Mov Disord. (2012) 27:617–26. doi: 10.1002/mds.24996, PMID: 22508280

[3] Driver JA Logroscino G Gaziano JM Kurth T. Incidence and remaining lifetime risk of parkinson disease in advanced age. Neurology. (2009) 72:432–8. doi: 10.1212/01.wnl.0000341769.50075.bb, PMID: 19188574 PMC2676726

[4] Wang L Peng JL Yang JB Gan L Zeng S Wang HY. Effects of rhythmic auditory stimulation on gait and motor function in Parkinson's disease: a systematic review and meta-analysis of clinical randomized controlled studies. Front Neurol. (2022) 13:818559. doi: 10.3389/fneur.2022.818559 35493833 PMC9053573

[5] Cochen DC Dotov DG Ihalainen P Bégel V Galtier F Lebrun C . Rhythmic abilities and musical training in Parkinson’s disease: do they help? NPJ Park Dis. (2018) 4:8. doi: 10.1038/s41531-018-0043-7 PMC5865140 29582000

[6] Calabrò RS Naro A Filoni S Pullia M Billeri L Tomasello P . Walking to your right music: a randomized controlled trial on the novel use of treadmill plus music in Parkinson’s disease. J Neuroeng Rehabil. (2019) 16:68. doi: 10.1186/s12984-019-0533-9, PMID: 31174570 PMC6555981

[7] Bukowska AA Krężałek P Mirek E Bujas P Marchewka A. Neurologic music therapy training for mobility and stability rehabilitation with Parkinson's disease-a pilot study. Front Hum Neurosci. (2016) 9:710. doi: 10.3389/fnhum.2015.00710 26858628 PMC4726780

[8] Bella SD Benoit CE Farrugia N Schwartze M Kotz SA. Effects of musically cued gait training in Parkinson’s disease: beyond a motor benefit. Ann N Y Acad Sci. (2015) 1337:77–85. doi: 10.1111/nyas.12651, PMID: 25773620

[9] Wu ZL Kong LY Zhang QX. Research progress of music therapy on gait intervention in patients with Parkinson’s disease. Int J Environ Res Public Health. (2022) 19:9568. doi: 10.3390/ijerph19159568, PMID: 35954925 PMC9368619

[10] Thaut MH McIntosh GC Rice RR Miller RA Rathbun J Brault JM. Rhythmic auditory stimulation in gait training for Parkinson’s disease patients. Mov Disord. (1996) 11:193–200. 8684391 10.1002/mds.870110213

[11] Pohl P Wressle E Lundin F Miller RA Rathbun J Brault JM. Group-based music intervention in Parkinson's disease - findings from a mixed-methods study. Clin Rehabil. (2020) 34:533–44. doi: 10.1177/0269215520907669 32070122 PMC7132435

[12] Harrison EC Horin AP Earhart GM. Mental singing reduces gait variability more than music listening for healthy older adults and people with Parkinson disease. J Neurol Phys Ther. (2019) 43:204–11. doi: 10.1097/NPT.0000000000000288, PMID: 31449178 PMC6744333

[13] Satoh M Kuzuhara S. Training in mental singing while walking improves gait disturbance in Parkinson’s disease patients. Eur Neurol. (2008) 60:237–43. doi: 10.1159/000151699, PMID: 18756088

[14] Sotomayor MJM Giráldez VA Rico GR. Music therapy and Parkinson's disease: a systematic review from 2015-2020. Int J Environ Res Public Health. (2021) 18:11618. doi: 10.3390/ijerph182111618 34770129 PMC8582661

[15] Stegemöller EL Zaman A Shelley M Patel B Kouzi AE Shirtcliff EA. The effects of group therapeutic singing on cortisol and motor symptoms in persons with Parkinson's disease. Front Hum Neurosci. (2021) 15:703382. doi: 10.3389/fnhum.2021.703382, PMID: 34381345 PMC8349974

[16] Pacchetti C Mancini F Aglieri R Fundarò C Martignoni E Nappi G. Active music therapy in Parkinson’s disease: an integrative method for motor and emotional rehabilitation. Psychosom Med. (2000) 62:386–93. doi: 10.1097/00006842-200005000-00012, PMID: 10845352

[17] Lee HJ Ko B. Effects of music-based interventions on motor and non-motor symptoms in patients with Parkinson’s disease: a systematic review and meta-analysis. Int J Environ Res Public Health. (2023) 20:1046. doi: 10.3390/ijerph20021046, PMID: 36673802 PMC9859027

[18] Page MJ Moher D Bossuyt PM Boutron I Hoffmann TC Mulrow CD . PRISMA 2020 explanation and elaboration: updated guidance and exemplars for reporting systematic reviews. BMJ. (2021) 372:160. doi: 10.1136/bmj.n160, PMID: 33781993 PMC8005925

[19] Higgins JPT Savovi J Page MJ Elbers RG Sterne JAC. Assessing risk of bias in a randomized trial. Cochrane handbook for systematic reviews of interventions. Hoboken, NJ, USA: John Wiley & Sons, Ltd. (2021).

[20] Janzen TB Haase M Thaut MH. Rhythmic priming across effector systems: a randomized controlled trial with Parkinson's disease patients. Hum Mov Sci. (2019) 64:355–65. doi: 10.1016/j.humov.2019.03.001, PMID: 30852469

[21] Murgia M Pili R Corona F Sors F Agostini TA Bernardis P . The use of footstep sounds as rhythmic auditory stimulation for gait rehabilitation in Parkinson's disease: a randomized controlled trial. Front Neurol. (2018) 9:348. doi: 10.3389/fneur.2018.00348, PMID: 29910764 PMC5992388

[22] Capato TTC Nonnekes J de Vries NM IntHout J Barbosa ER Bloem BR. Effects of multimodal balance training supported by rhythmical auditory stimuli in people with advanced stages of Parkinson's disease: a pilot randomised clinical trial. J Neurol Sci. (2020) 418:117086. doi: 10.1016/j.jns.2020.117086, PMID: 32891018

[23] Thaut MH Rice RR Braun JT Hurt-Thaut CP McIntosh GC. Rhythmic auditory stimulation for reduction of falls in Parkinson's disease: a randomized controlled study. Clin Rehabil. (2019) 33:34–43. doi: 10.1177/0269215518788615 30033755

[24] Song JH Zhou PY Cao ZH Ding ZG Chen HX Zhang GB. Rhythmic auditory stimulation with visual stimuli on motor and balance function of patients with Parkinson's disease. Eur Rev Med Pharmacol Sci. (2015) 19:2001–7. 26125261

[25] Kadivar Z Corcos DM Foto J Hondzinski JM. Effect of step training and rhythmic auditory stimulation on functional performance in Parkinson patients. Neurorehabil Neural Repair. (2011) 25:626–35. doi: 10.1177/1545968311401627, PMID: 21436393

[26] Li KP Zhang ZQ Zhou ZL Su J Wu X Shi B . Effect of music-based movement therapy on the freezing of gait in patients with Parkinson’s disease: a randomized controlled trial. Front Aging Neurosci. (2022) 14:924784. doi: 10.3389/fnagi.2022.924784, PMID: 36337701 PMC9627030

[27] Li F Wang D Ba X Liu Z Zhang M. The comparative effects of exercise type on motor function of patients with Parkinson’s disease: a three-arm randomized trial. Front Hum Neurosci. (2022) 16:1033289. doi: 10.3389/fnhum.2022.1033289, PMID: 36530197 PMC9751317

[28] Bradt J. Randomized controlled trials in music therapy: guidelines for design and implementation. J Music Ther. (2012) 49:120–49. doi: 10.1093/jmt/49.2.120, PMID: 26753215

[29] Mollinedo I Cancela JM. Evaluation of the psychometric properties and clinical applications of the timed up and go test in Parkinson disease: a systematic review. J Exerc Rehabil. (2020) 16:302–12. doi: 10.12965/jer.2040532.266, PMID: 32913835 PMC7463070

[30] Mollà-Casanova S Pedrero-Sánchez J Inglés M Lopez-Pascual J Munoz-Gomez E Aguilar-Rodríguez M . Impact of Parkinson’s disease on functional mobility at different stages. Front Aging Neurosci. (2022) 14:935841. doi: 10.3389/fnagi.2022.935841, PMID: 35783141 PMC9249436

[31] Koshimori Y Thaut MH. Future perspectives on neural mechanisms underlying rhythm and music based neurorehabilitation in Parkinson’s disease. Ageing Res Rev. (2018) 47:133–9. doi: 10.1016/j.arr.2018.07.001, PMID: 30005957

[32] Ghai S Ghai I Schmitz G Effenberg AO. Effect of rhythmic auditory cueing on parkinsonian gait: a systematic review and meta-analysis. Sci Rep. (2018) 8:506. doi: 10.1038/s41598-017-16232-5, PMID: 29323122 PMC5764963

[33] McIntosh GM Brown SH Rice RR. Rhythmic auditory-motor facilitation of gait patterns inpatients with Parkinson’s disease. J Neurol Neurosurg Psychiatry. (1997) 62:22–6. 9010395 10.1136/jnnp.62.1.22 PMC486690

[34] Rubinstein TC Giladi N Hausdorff JM. The power of cueing to circumvent dopamine deficits: a review of physical therapy treatment of gait disturbances in Parkinson’s disease. Mov Disord. (2002) 17:1148–60. doi: 10.1002/mds.10259, PMID: 12465051

[35] Thaut MH. The future of music in therapy and medicine. Ann N Y Acad Sci. (2005) 1060:303–8. doi: 10.1196/annals.1360.023, PMID: 16597779

[36] Wei Y Qiao Z. Neurologic music therapy's impact on neurological disorders. J Neurosci Res. (2024) 102:e70000. doi: 10.1002/jnr.70000, PMID: 39625180

[37] Altenmüller E Schlaug G. Neurologic music therapy: the beneficial effects of music making on neurorehabilitation. Acoust Sci Technol. (2013) 34:5–12. doi: 10.1250/ast.34.5

[38] Thaut MH McIntosh GC Hoemberg V. Neurobiological foundations of neurologic music therapy: rhythmic entrainment and the motor system. Front Psychol. (2015) 5:1185. doi: 10.3389/fpsyg.2014.01185, PMID: 25774137 PMC4344110

[39] Jauset-Berrocal JA Soria-Urios G. Cognitive neurorehabilitation: the foundations and applications of neurologic music therapy. Rev Neurol. (2018) 67:303–10. doi: 10.33588/rn.6708.2018021 30289154

[40] Braunlich K Seger CA Jentink KG Buard I Kluger BM Thaut MH. Rhythmic auditory cues shape neural network recruitment in Parkinson's disease during repetitive motor behavior. Eur J Neurosci. (2019) 49:849–58. doi: 10.1111/ejn.14227, PMID: 30375083 PMC6426668

[41] Haro-Martínez AM Lubrini G Madero-Jarabo R Díez-Tejedor E Fuentes B. Melodic intonation therapy in post-stroke nonfluent aphasia: a randomized pilot trial. Clin Rehabil. (2019) 33:44–53. doi: 10.1177/0269215518791004, PMID: 30056747

[42] Behaghel E Zumbansen A. Singing for the rehabilitation of acquired neurogenic communication disorders: continuing the evidence dialogue with a survey of current practices in speech-language pathology. Health. (2022) 10:1010. doi: 10.3390/healthcare10061010, PMID: 35742061 PMC9222374

[43] Zhang X Li J Du Y. Melodic intonation therapy on non-fluent aphasia after stroke: a systematic review and analysis on clinical trials. Front Neurosci. (2022) 15:753356. doi: 10.3389/fnins.2021.753356, PMID: 35153655 PMC8829877

